# Restoration of atypical protein kinase C ζ function in autosomal dominant polycystic kidney disease ameliorates disease progression

**DOI:** 10.1073/pnas.2121267119

**Published:** 2022-07-22

**Authors:** Masaw Akbari, Jonathan D. West, Nicholas Doerr, Kevin R. Kipp, Neda Marhamati, Sabrina Vuong, Yidi Wang, Markus M. Rinschen, Jeffrey J. Talbot, Oliver Wessely, Thomas Weimbs

**Affiliations:** ^a^Department of Molecular, Cellular, and Developmental Biology; and Neuroscience Research Institute, University of California, Santa Barbara, CA 93106-9625;; ^b^Department II of Internal Medicine and Center for Molecular Medicine Cologne, University of Cologne, Faculty of Medicine and University Hospital Cologne, 50931 Cologne, Germany;; ^c^Department of Cardiovascular & Metabolic Sciences, Lerner Research Institute, Cleveland Clinic Foundation, Cleveland, OH 44195

**Keywords:** polycystic kidney disease, polycystin-1, protein kinase C ζ

## Abstract

Autosomal dominant polycystic kidney disease (ADPKD) affects more than 500,000 individuals in the United States alone. In most cases, ADPKD is caused by a loss-of-function mutation in the *PKD1* gene, which encodes polycystin-1 (PC1). Previous studies reported that PC1 interacts with atypical protein kinase C (aPKC). Here we show that PC1 binds to the ζ isoform of aPKC (PKCζ) and identify two PKCζ phosphorylation sites on PC1’s C-terminal tail. PKCζ expression is down-regulated in patients with ADPKD and orthologous and nonorthologous PKD mouse models. We find that the US Food and Drug Administration–approved drug FTY720 restores PKCζ expression in in vitro and in vivo models of polycystic kidney disease (PKD) and this correlates with ameliorated disease progression in multiple PKD mouse models. Importantly, we show that FTY720 treatment is less effective in PKCζ null versions of these PKD mouse models, elucidating a PKCζ-specific mechanism of action that includes inhibiting STAT3 activity and cyst-lining cell proliferation. Taken together, our results reveal that PKCζ down-regulation is a hallmark of PKD and that its stabilization by FTY720 may represent a therapeutic approach to the treat the disease.

## Significance

Autosomal dominant polycystic kidney disease (ADPKD) is a genetic disorder commonly caused by mutations in polycystin-1. The disease is associated with severe morbidity and has limited therapeutic options, with most patients requiring dialysis or transplantation by the sixth decade. Our work adds to understanding polycystic kidney disease (PKD) pathogenesis by clarifying the role of PKCζ in ADPKD and by presenting PKCζ as a potential therapeutic target. We show that PKCζ phosphorylates polycystin-1 at two specific serine residues. We demonstrate that PKCζ is aberrantly down-regulated in human ADPKD and mouse models of PKD and that its activity can be restored via treatment with the US Food and Drug Administration–approved drug FTY720. Last, we demonstrate that FTY720 treatment ameliorates disease progression in PKD mouse models and that these improvements are dependent on PKCζ expression.

Autosomal dominant polycystic kidney disease (ADPKD) is the most common genetic kidney disease, affecting more than 12 million people worldwide. The cause is linked to mutations in either the *PKD1* or *PKD2* gene. Disease progression involves the formation of numerous fluid-filled cysts throughout both kidneys that progressively enlarge, replacing the normal renal parenchyma and eventually causing end-stage renal disease in most patients with ADPKD prior to their sixth decade (1, 2). ADPKD is a disease for which there remains an urgent need for treatment. Currently, tolvaptan is the only US Food and Drug Administration (FDA)-approved drug for ADPKD. Tolvaptan treatment slows, but does not prevent, disease progression. Patient access to tolvaptan is limited due to prescription criteria, extremely high cost, significant side effects, and the potential for toxicities. Most patients with ADPKD who are receiving tolvaptan still eventually require renal transplantation or dialysis (3, 4).

The *PKD1* gene encodes polycystin-1 (PC1), an ∼500-kDa glycoprotein comprising a large extracellular domain, 11 transmembrane domains, and a short cytoplasmic tail (5). The *PKD2* gene encodes polycystin-2 (PC2), a 130-kDa, nonselective cation channel of the TRP family (6). PC1 and PC2 interact via their coiled-coil motifs in their C-terminal tails and are thought to form a mechanically sensitive cation channel (7). However, numerous other functions have been ascribed to these proteins, and the actual purpose of the polycystins is not well understood (1). PC1 has been shown to localize to several cellular compartments, including primary cilia, cell–cell junctions, the endoplasmic reticulum, and the nucleus (8–11). PC1’s diversity of subcellular localizations supports its involvement in many complex intracellular signaling pathways. Within the PC1 sequence, most of the reported protein interactions map to its ∼200-residue C-terminal cytoplasmic tail.

Our laboratory and others have shown that PC1 regulates STAT3 and mTOR signaling (12–23), both of which are aberrantly activated in rodent models of polycystic kidney disease (PKD) and human ADPKD. STAT3 is shown to be strongly activated in cyst-lining epithelial cells in human ADPKD (15) and in several PKD rodent models (15, 17, 18, 24, 25), compared with normal kidneys. Initial inhibitor studies suggest that aberrant STAT3 activation may be a driving force of renal cyst growth (17, 18, 26); however, these inhibitors exhibit limited specificity and off-target effects.

The PC1 C-terminal tail interacts with several kinases in vitro, including PKA (27), PRKX (27), and Src (28). PC1 also interacts with the tyrosine kinase JAK2 regulating PC1-dependent STAT3 activation (15). We previously reported the interaction of PC1 with atypical protein kinase C (aPKC), as exemplified by PKCζ (29), and another group demonstrated a functional interaction between PC1 and aPKC to mediate polarized cell migration during embryonic renal development (30). They reported that directional cell division is disrupted in mouse models of PKD due to dysregulation of the Par3/6 polarity complexes.

The aPKC family, which includes isoforms ζ (PKCζ) and λ/ι (PKCλ/ι), require neither calcium nor diacylglycerol for activation (31). PKCζ is known for its role in epithelial cell polarity, ciliogenesis, metabolism, and calcium signaling, as well as in various signaling pathways, including NF-κB (32, 33), AMPK (34), and S6K (35–38). All of these pathways are also dysregulated in ADPKD, but it is not known whether PKCζ is involved in this dysregulation.

In this study, we demonstrate that PKCζ interacts with the C-terminal cytoplasmic tail of PC1, consistent with the findings of Castelli et al. (30). We further show that PKCζ can directly phosphorylate the PC1 tail, and we identify the phosphorylation sites. We investigate the role of PKCζ in ADPKD progression and report that PKCζ levels are reduced in both mouse ADPKD models and human ADPKD. We show that while transgenic PKCζ knockout does not further accelerate disease progression in PKD mouse models, activating the protein via treatment with the FDA-approved drug FTY720 (fingolimod) significantly ameliorates multiple disease markers in PKD mouse models. Last, we show that FTY720 treatment is less effective in PKCζ knockout versions of our PKD mouse models, suggesting some PKCζ-specific mechanisms of action.

Taken together, these results indicate that PKCζ is dysregulated in ADPKD and that its pharmacological activation can alleviate disease burden, thus presenting a therapeutic approach to ADPKD.

## Results

### PKCζ Interacts with PC1 via its C-Terminal Tail.

**Fig. 1.**
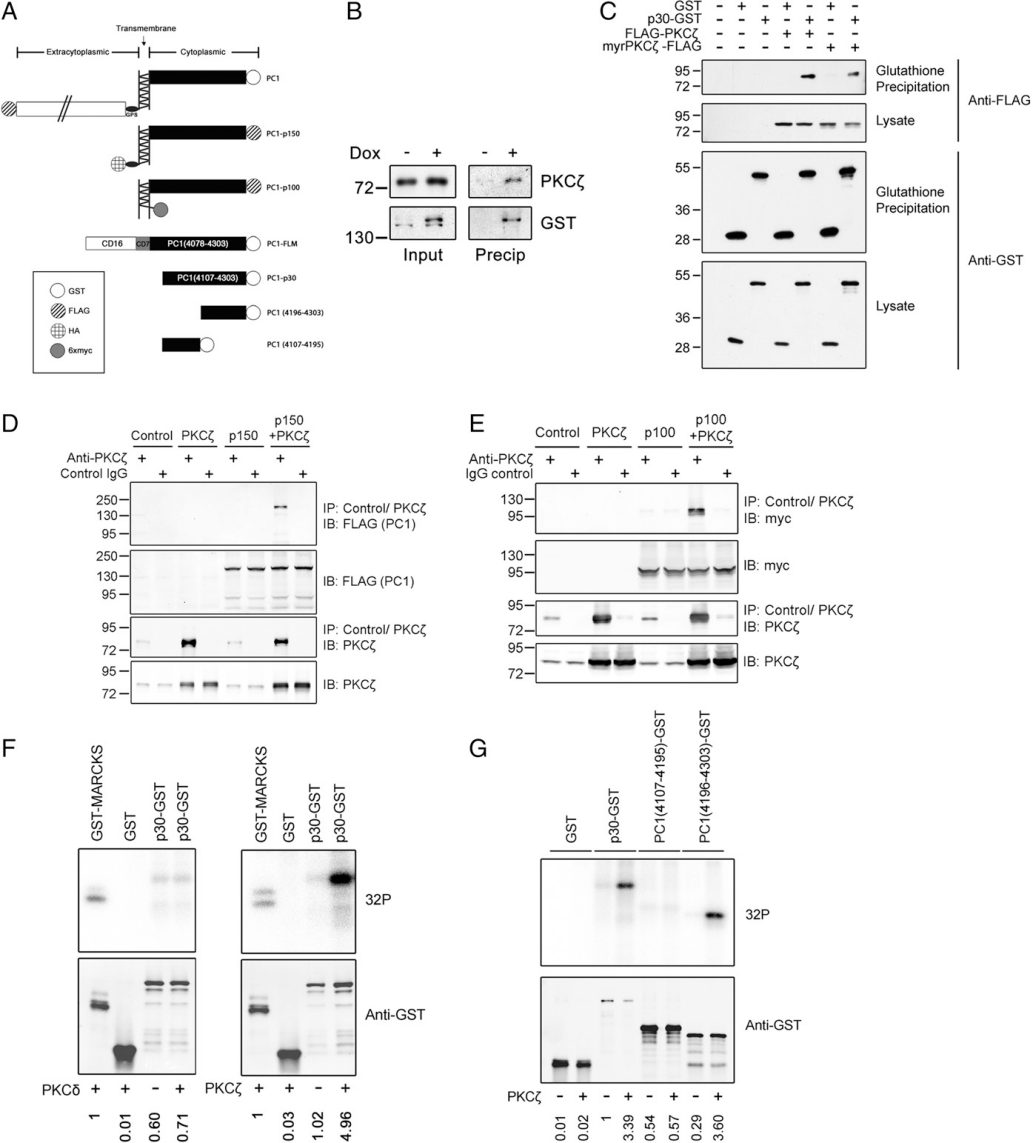
PKCζ interacts with and phosphorylates PC1 via its C-terminal tail. (*A*) Schematic of PC1-expression constructs. (*B*) MDCK PC1-GST Tet-On cells were grown in the presence of 50 ng/mL DOX. Lysates were precipitated with glutathione-agarose and Western blots were carried out using the indicated antibodies. (*C*) HEK293T cells transiently expressing PC1-p30-GST or GST were cotransfected with FLAG-PKCζ or constitutively active myr-PKCζ-FLAG. Lysates were precipitated with glutathione-agarose and analyzed by Western blot. (*D* and *E*) HEK293T cells transfected with empty vector (control), PKCζ, PC1-p150-FLAG (*D*), or myc-PC1-p100 (*E*) were lysed and subjected to immunoprecipitation with anti-PKCζ, or a control IgG. Lysates and precipitates were analyzed by Western blot. (*F*, *G*) GST, PC1-p30-GST, PC1-p30 (4107-4195)-GST, and PC1-p30 (4196-4303)-GST, were purified from HEK293T cell lysates with glutathione-agarose. Precipitated proteins were incubated with recombinant PKCζ or PKCδ for 45 min. Phosphorylated proteins were resolved by sodium dodecyl sulfate–polyacrylamide gel electrophoresis and detected by autoradiography. Protein loading was determined in parallel by Western blot. Phospho-signals were normalized to protein loading. Relative phospho-signal is indicated below each autoradiograph. The general PKC substrate GST-MARCKS(96-184) was used as a positive control. IB, immunoblot; IP, immunoprecipitation.

Phosphorylated PKCζ has been reported to bind to PC1 and regulate the Par3/Par6/aPKC complex (30). We had also independently uncovered this interaction between PC1 and PKCζ (29) and further investigated it by seeking to confirm the interaction between full-length PC1 and endogenous PKCζ in renal epithelial cells. To do this, a stable Madin-Darby canine kidney (MDCK) cell line was generated that expresses a previously described epitope-tagged form of full-length PC1 under the control of a doxycycline (DOX)-inducible promoter (MDCK-PC1-GST^Tet-On^; Fig. 1*A*) (39). Coprecipitation experiments indicated that PKCζ forms a stable complex with PC1 under these conditions (Fig. 1*B*), consistent with the findings of Castelli et al. (30).

To identify the minimal region of PC1 required to interact with PKCζ, expression constructs were generated encoding full-length or several truncated forms of PC1, fused to a C-terminal glutathione-*S*-transferase (GST) tag (Fig. 1*A*). To verify the utility of our constructs, we coprecipitated the C-terminal cytoplasmic PC1 tail (PC1-p30-GST) and PC2, a well-validated binding partner of the PC1 cytoplasmic tail (40, 41), and found that PC2 interacts with PC1-p30-GST, but not GST alone (*SI Appendix*, Fig. S1). Using this approach, we found that PC1-p30-GST interacts with PKCζ (Fig. 1*C*). This interaction was unaltered by coexpression of a constitutively active PKCζ mutant (myr-PKCζ), suggesting that the interaction is not enhanced by PKCζ activation. We next tested whether PKCζ binds to other biologically relevant forms of PC1, by coexpressing several naturally occurring and functionally distinct PC1 proteolytic fragments with PKCζ in human embryonic kidney 293T (HEK293T) cells (Fig. 1*A*). We demonstrate that PKCζ interacts with both the ∼150-kDa C-terminal fragment (PC1-p150) (Fig. 1*D*), which results from cleavage of the G protein–coupled receptor proteolytic site (39), and the ∼100-kDa C-terminal fragment (PC1-p100) (Fig. 1*E*), which reportedly localizes to the endoplasmic reticulum and regulates store-operated calcium channels (42).

Altogether, these results demonstrate that PC1 interacts with PKCζ, that the C-terminal cytoplasmic tail of PC1 is sufficient for the interaction, and that this interaction can occur whether PC1 is membrane anchored (full-length PC1, PC1-p150, and PC1-p100) or soluble (PC1-p30).

### PKCζ Phosphorylates the C-Terminal Tail of PC1.

To investigate the ability of PKCζ to phosphorylate PC1, we conducted in vitro kinase experiments in which PC1-p30-GST was transiently expressed and purified from mammalian cells and incubated with recombinant PKCζ in the presence of ^32^P-ATP (32). We found that PKCζ caused strong phosphorylation of PC1-p30-GST, but not GST alone, and that PC1-p30-GST was not phosphorylated by the novel PKC isoform PKCδ (Fig. 1*F*), indicating that the observed activity is specific to PKCζ. We verified the activity of our kinases by showing that GST-MARCKS, a known substrate of most PKC isoforms (43), was phosphorylated by both PKCζ and PKCδ (*SI Appendix*, Fig. S2).

We then attempted to determine the location of PKCζ-mediated PC1 tail phosphorylation. To identify the region of the PC1 tail in which phosphorylation occurs, we used truncation mutants containing either the N-terminal half [PC1(4107-4196)-GST] or the C-terminal half [PC1(4196-4303)-GST] of the PC1 tail (Fig. 1*A*) and found that PKCζ primarily phosphorylates the C-terminal half (Fig. 1*G*). To identify the specific phosphorylation site(s) within the PC1 tail, we first used experimentally validated algorithms to computationally identify several sites as potential PKCζ phospho-sites (*SI Appendix*, Fig. S3*A*) (44, 45). We then coexpressed PC1-p30-GST with either the constitutively kinase-active PKCζ (T410E) or the kinase-dead PKCζ (T410A) mutant, enriched the phosphopeptides from cell lysates, and analyzed them by nano-liquid chromatography–tandem mass spectrometry. Using this approach, we identified three sites within the PC1 tail that are phosphorylated when coexpressed with active, but not inactive, PKCζ (*SI Appendix*, Fig. S3*B* and *C*). Notably, all of these sites were in agreement with the phosphosite prediction tool (*SI Appendix*, Fig. S3*A*). Of the three identified sites, two (4258/59 and 4263) are located within the C-terminal half and one is located within the N-terminal half of the PC1 tail (4165/4166). Identification of the C-terminal sites is consistent with our in vitro phosphorylation assay (Fig. 1*G*), whereas the mass spectrometry approach was necessary to identify the N-terminal site. Two additional phosphorylation sites (S4213 and T4285) were found to be constitutively phosphorylated (*SI Appendix*, Fig. S3*C*) independent of PKCζ, suggesting that these residues are targets of different kinases and consistent with the finding that these sites were not identified computationally (*SI Appendix*, Fig. S3*A*). Altogether, these results demonstrate that PKCζ can bind and phosphorylate the PC1 cytosolic tail in vitro. Previous work by Castelli et al. (30) shows that PC1 interacts with phosphorylated PKCζ in vivo, suggesting that the kinase is active in this complex and that these phosphosites may be physiologically relevant.

### PKCζ Expression Is Down-Regulated in the Kidneys of Patients with ADPKD and in PKD Mouse Models.

After confirming and further characterizing the interaction between PC1 and PKCζ, our next goal was to better understand how PKCζ signaling is altered in PKD. Previous work has shown that directional cell division is disrupted in mouse models of PKD due to dysregulation of the Par3/6 polarity complexes (30). However, in this prior study, regulation at the level of PKCζ activity and expression was not fully explored, nor was the effect of modulating PKCζ function on PKD disease progression. The previous investigation of in vivo PKCζ expression suggested up-regulation in cystic kidneys (30). However, according to the manufacturer’s data sheet, the antibody used in that study cannot differentiate between the aPKC ζ and ι/λ isoforms, due to sequence homology. Given this limitation, we elected to use isoform-specific antibodies when assessing the expression of aPKC isoforms.

**Fig. 2.**
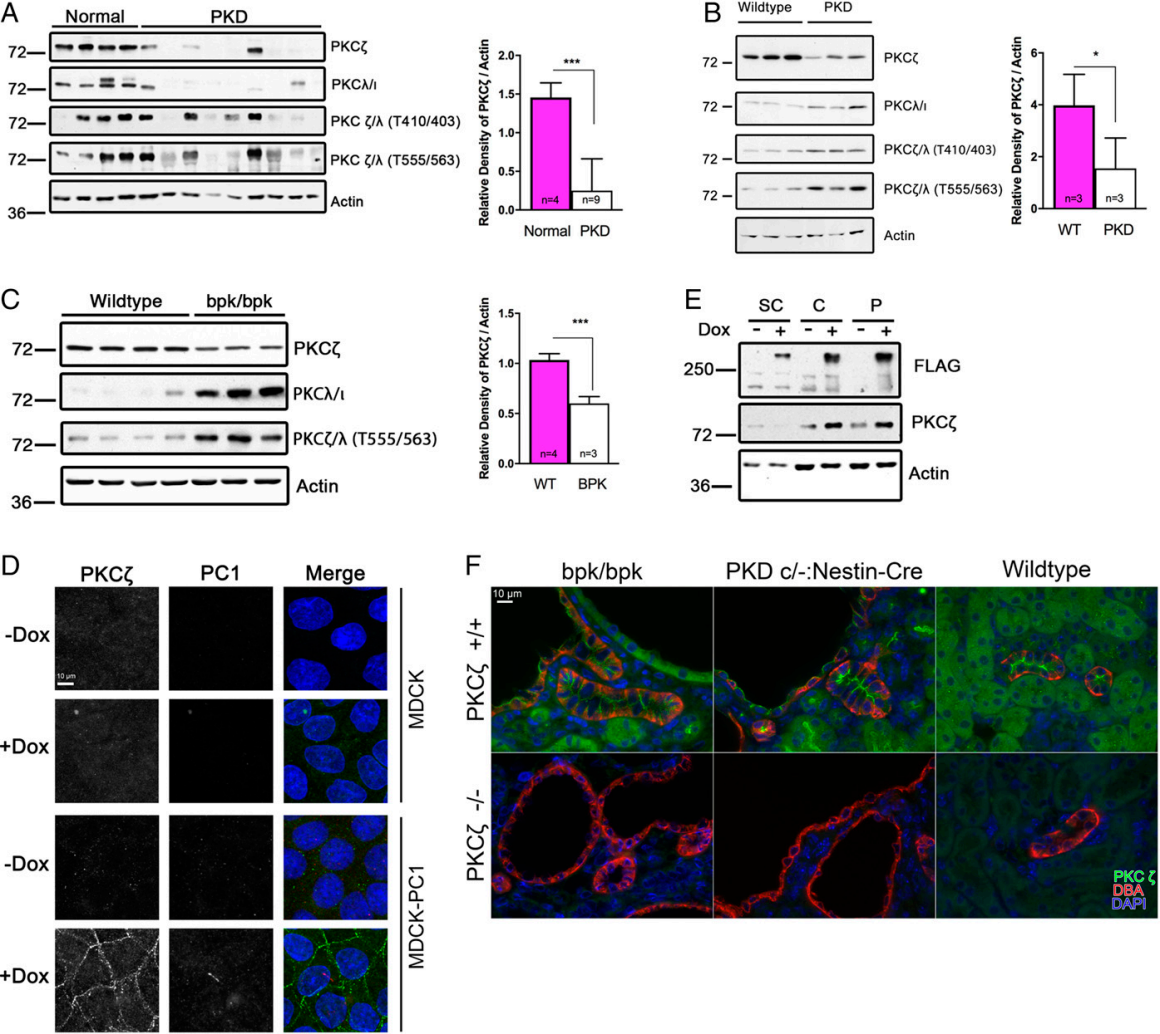
PKCζ expression is dysregulated in PKD. (*A*) Western blots of kidney tissue lysates from normal or patients with ADPKD. (*B*) Western blots of kidney tissue lysates from postnatal day 49 Pkd1^cond/cond^ (PKD) or wild-type (WT) mice. (*C*) Western blots of kidney-tissue lysates from postnatal day 17 bpk/bpk or wild-type mice. (*D*) Immunofluorescence micrographs of polarized MDCK cells or MDCK-PC1-myc^Tet-On^ cells grown with or without 50 ng/mL DOX. PC1 was detected with an anti-myc tag antibody (red). PKCζ (green) and nuclei (blue) staining was also performed. Scale bar, 10 μm. (*E*) Western blot of subconfluent (SC), confluent (C), or postconfluent (P) MDCK-PC1-FLAG^Tet-On^ cells grown with or without 50 ng/mL DOX. (*F*) Immunofluorescence staining of PKCζ (green), DBA marker of renal collecting ducts (red) and nuclei (blue) 5-μm kidney sections from cystic (bpk/bpk, PKD) and wild-type mice. Scale bar, 10 μm. *P < 0.05; ***P < 0.001.

By immunoblot analysis, we observed decreased PKCζ expression in kidney-tissue lysates from patients with ADPKD, compared with normal controls (Fig. 2*A*). We also observed decreased PKCζ expression in cystic kidney lysates of both an orthologous ADPKD mouse model (Pkd1^cond/cond^) (Fig. 2*B*) and the nonorthologous bpk mouse model (bpk/bpk) (Fig. 2*C*), as compared with control mice. We observed a similar down-regulation in PKCζ expression in a retinal pigment epithelial (RPE1) cell line with a stable knockdown of PC1 expression using Pkd1 shRNA (*SI Appendix*, Fig. S4*A*). This relationship was further supported by a DOX-inducible PC1-expressing cell line, which showed that inducible expression of PC1 increased PKCζ expression. This effect was also observed by immunofluorescence microscopy in polarized MDCK cells (Fig. 2*D*) and by immunoblot in confluent and postconfluent MDCK cells (Fig. 2*E*). Interestingly, we did not observe colocalization of the two proteins by fluorescent microscopy. As PC1 expression levels are much lower than PKCζ levels, this may suggest a nonstochiometric relationship between the two proteins. Thus, we hypothesize that this interaction is likely transient, perhaps only necessary for initial establishment of the stabilized par3-PKCζ polarity complex that was previously reported (30). Regardless, these collective findings suggest that proper expression of functional PC1 plays a role in maintaining PKCζ expression.

The activity of atypical PKCs is regulated by phosphorylation at two loci. Unexpectedly, we noticed an increase in total phosphorylated aPKC (PKCζ and PKC ι/λ) at both the T555/563 and T410/403 sites in orthologous (Fig. 2*B*) and nonorthologous (Fig. 2*C*) mouse models. Importantly, again these antibodies were unable to distinguish between the two closely related aPKC isoforms, PKCζ and PKCι/λ. Given our finding that PKCζ expression is down-regulated (Fig. 2*B* and *C*), we attributed the increase in aPKC phosphorylation to the up-regulation of activated PKCι/λ. This explanation is supported by the fact that there is little difference in levels of phosphorylated aPKC in bpk mice and those that are also PKCζ knockouts (*SI Appendix*, Fig. S5). Furthermore, we observed an up-regulation in PKCι/λ expression in RPE1 cells with a stable PC-1 knockdown (*SI Appendix*, Fig. S4*A*), and both orthologous (Fig. 2*B*) and nonorthologous (Fig. 2*C*) mouse models. Together, these findings suggest a compensatory mechanism between the two aPKC isoforms and that PC1 has a direct effect on the balance of these two proteins. While these findings warrant further investigation of both isoforms as potential treatment approaches, we elected to focus the remainder of our investigation on PKCζ.

We next investigated the localization of PKCζ in situ using immunofluorescence microscopy on kidney sections. In wild-type mouse kidneys, PKCζ localizes diffusely in the cytoplasm of tubule cells, except in collecting duct/distal tubule cells, where it localizes very distinctly to apical junctions (Fig. 2*F*). Kidneys from PKCζ-null mice served as negative controls. Costaining with the renal collecting duct marker *Dolichos biflorus* agglutinin (DBA) revealed that PKCζ most frequently localizes to the apical junctions of distal tubule epithelial cells in both wild-type and cystic mouse kidneys (Pkd1^cond/−^ and bpk mouse models) (Fig. 2*F*), consistent with the known cellular origin of these cysts (20). Generally, we observed no difference in the localization of PKCζ between wild-type and cystic mouse kidneys, especially in normal renal tubules, although there appeared to be less regular localization and expression levels in the cyst-lining cells (Fig. 2*F*).

### PKCζ Knockout Does Not Significantly Alter Disease Progression in Multiple Mouse Models of PKD.

**Fig. 3.**
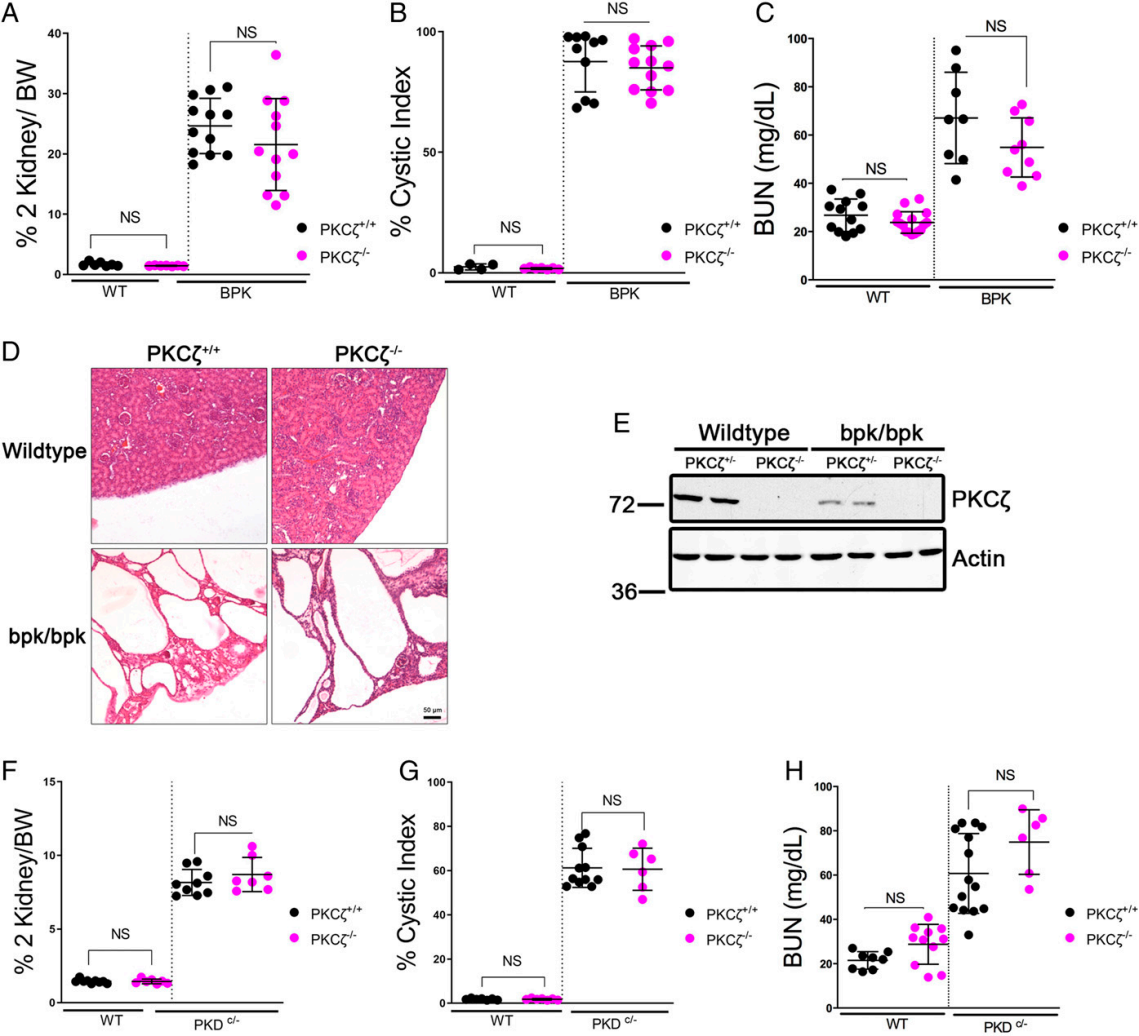
PKCζ knockout does not significantly affect disease progression in the bpk or ADPKD orthologous Pkd1^cond/−^ mouse models of PKD. PKCζ-null mice were crossed into the bpk mouse model and assessed for various markers of disease at day 17, including (*A*) two-kidney to body weight ratio, (*B*) cystic index, and (*C*) BUN. (*D*) Representative 10× images of H&E-stained kidney sections from mice with the indicated genotypes. (*E*) Western blot of mouse kidney tissue from wild-type (WT) and bpk/bpk (bpk) mice either expressing or not expressing PKCζ. PKCζ-null mice were crossed into the Pkd1^cond/-^ mouse model and assessed for markers of disease at postnatal day 21, including (*F*) two-kidney to body weight (%) ratio, (*G*) cystic index, and (*H*) BUN (mg/dL). BW, body weight; NS, not significant.

Given our findings that PKCζ expression is aberrantly down-regulated in both human ADPKD and multiple PKD mouse models, we next investigated the potential effect of a PKCζ knockout on disease progression. We crossed a transgenic PKCζ knockout (PKCζ^−/−^) into the bpk and Pkd1^cond/−^ mouse models, respectively, and found that this alteration had no significant effect on disease progression in either model (Fig. 3). We verified the PKCζ knockout by PCR and Western blot (Fig. 3*E*). In the bpk model, we found no significant changes in the two-kidney to body weight ratio (Fig. 3*A*), cystic index (Fig. 3*B*), or blood urea nitrogen (BUN) concentration (Fig. 3*C*) in PKCζ^−/−^ mice (bpk/bpk:PKCζ^−/−^), as compared with unaltered bpk mice (bpk/bpk:PKCζ^+/+^). Representative hematoxylin and eosin (H&E)-stained kidney sections (Fig. 3*D*) display similar morphology changes in both mouse strains. In the Pkd1^cond/−^ model, we also found that disease progression was similar at postnatal day 21 when comparing PKCζ^−/−^ mice (Pkd1^cond/−^:PKCζ^−/−^) with unaltered Pkd1^cond/−^ mice (Pkd1^cond/−^:PKCζ^+/+^). Specifically, we found that there were no significant changes in the two-kidney to body weight ratio (Fig. 3*F*), cystic index (Fig. 3*G*), or BUN concentration (Fig. 3*H*). Our finding that complete loss of PKCζ expression does not further accelerate disease progression suggests that the residual PKCζ in these tissues is insufficient to ameliorate cystogenesis in these two very rapidly progressing disease models, and that perhaps cyst formation and growth are already near maximal.

### FTY720 Activates PKCζ In Vitro and In Vivo.

Since we found that a PKCζ knockout had no effect on disease progression in our PKD mouse models, we hypothesized that restoring PKCζ activity and expression may slow cyst formation. FTY720 (fingolimod) is a first-in-class, small-molecule immunomodulatory drug that is FDA approved for use in relapsing multiple sclerosis (46). FTY720 has been shown to modulate ceramide levels (47), a well-established activator of PKCζ (48–50). Furthermore, once activated by sphingosine kinase 2, FTY720 acts as a structural analog of sphingosine-1-phosphate (S1P), which has been shown to activate PKCζ by directly binding to its kinase domain and relieving its autoinhibitory constraints (51).

Before proceeding with FTY720 treatment in vivo, we verified that FTY720 had the potential to modulate PKCζ function in culture. We first established that ceramide, indeed, activates PKCζ in kidney cells (*SI Appendix*, Fig. S6*A*) and in PC1-deficient RPE1 cells (*SI Appendix*, Fig. S6*B*). We then tested whether FTY720 can activate PKCζ in vitro. To achieve this, an RPE1 cell line was used in which PC1 expression was stably knocked down by short hairpin RNA (shRNA; PKD1 shRNA) (*SI Appendix*, Fig. S4*A*). Treatment of these cells with 250 nM FTY720 led to increased PKCζ activity over time (*SI Appendix*, Fig. S4*B*). FTY720 treatment led to similar PKCζ activation in the MDCK kidney cell line (*SI Appendix*, Fig. S4*E*).

We next investigated the signaling changes induced by a single injection of FTY720 in an orthologous mouse model of ADPKD (Pkd1^cond/−^). Single intraperitoneal (IP) injections of 3, 5, and 10 mg/kg FTY720 led to strong activation of PKCζ after 12 h compared with vehicle controls. No increase in the total PKCζ expression was observed at this time point in response to FTY720 treatment (*SI Appendix*, Fig. S4*C*); however, expression was increased after long-term treatment with the drug (*SI Appendix*, Fig. S4*D*).

### FTY720 Improves Disease Progression in Multiple Mouse Models of PKD in a PKCζ-Dependent Manner.

**Fig. 4.**
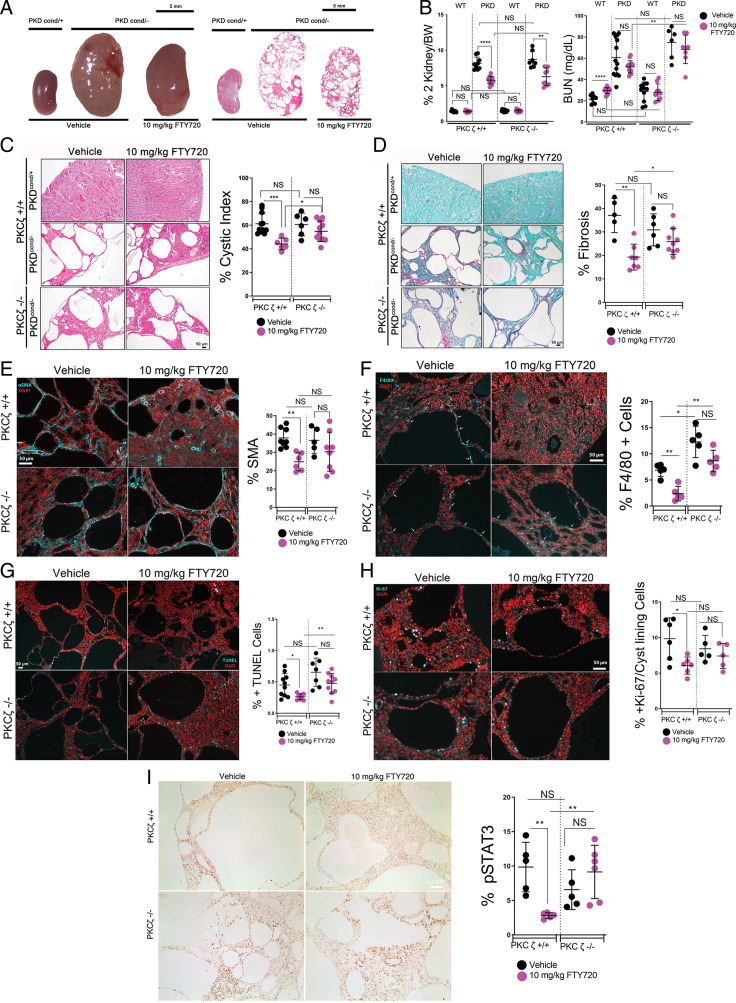
FTY720 improves disease progression in the ADPKD orthologous Pkd1^cond/−^ mouse model, while improvement is diminished in PKCζ knockout mice. Wild-type (WT) and Pkd1^cond/−^ mice (PKD^cond/−^), with and without a transgenic PKCζ knockout, were treated with daily IP injections of 10 mg/kg FTY720 or vehicle control from days 7 to 20 and assessed for disease progression. (*A*) Representative gross kidney images of wild-type and PKD vehicle or FTY720 treated (*Left*) and representative 4× images of full-kidney H&E-stained kidney sections (*Right*) (*B*) two-kidney to body weight ratio (% 2K/BW) (*Left*) and BUN (*Right*). (*C*) Representative images of H&E-stained wild-type, PKD, and PKD PKCζ-null mouse kidneys and percent cystic index. (*D*) Sirius red and fast-green collagen staining of PKD mouse kidneys and percent fibrosis. € α-SMA and quantification of PKD or PKD PKCζ knockout mice kidneys treated with 10 mg/kg FTY720 or vehicle injection. (*F*) Macrophage marker F4/80 immunofluorescence of PKD or PKD PKCζ-knockout mice kidneys treated with 10 mg/kg FTY720 or vehicle injection and quantification. (*G*) TUNEL assay for apoptosis of PKD and PKD PKCζ-null kidney sections and percentage of TUNEL-positive cells. (*H*) *K*_i_-67 cellular proliferation marker immunofluorescence stain and quantification performed by counting the cyst-lining–positive *K*_i_-67 cells per total cyst-lining cells. (*I*) Immunohistochemical stain for phospho-STAT3 (Y705) of PKD and PKD PKCζ-null kidney sections and quantification. Scale bars, 50 µm. NS, not significant. *P < 0.05; **P < 0.01.

Based on these results, we proceeded to treat the Pkd1^cond/−^ mouse model (PKD) with daily IP injections of 10 mg/kg FTY720 during the period of rapid disease progression (from postnatal days 7 to 20) and found that treatment improved various markers of disease severity. To determine whether these improvements in disease progression were mediated by the drug’s effect on PKCζ, we compared effects of identical treatment regimens on PKD mice that are wild type for PKCζ (PKD-PKCζ^+/+^), null for PKCζ (PKD-PKCζ^−/−^), or wild-type control mice. Treatment of cystic PKD-PKCζ^+/+^ mice with FTY720 led to overall reduction in renal cystic burden (Fig. 4*A*), reductions in both two-kidney to body weight ratio (Fig. 4*B*) and cystic index (Fig. 4*C*), compared with those treated with vehicle. Whereas the two-kidney to body weight ratio in FTY720-treated PKD-PKCζ^−/−^ mice still improved (Fig. 4*B*), there was no decrease in cystic index upon treatment (Fig. 4*C*). There was a nonsignificant trend toward decreased BUN in both PKD-PKCζ^+/+^ and PKD-PKCζ^−/−^ mice treated with FTY720, compared with those treated with vehicle (Fig. 4*B*). Changes in total body weight and other tissues in response to treatment were generally unremarkable (*SI Appendix*, Fig. S7).

Because renal interstitial fibrosis is a hallmark of PKD, we investigated whether FTY720 may affect collagen deposition. As shown in Fig. 4*D*, kidneys of PKD-PKCζ^+/+^ mice treated with FTY720 exhibited reduced collagen deposition compared with those of vehicle controls, significantly more so than in the kidneys of PKD-PKCζ^−/−^ mice. Myofibroblasts are largely responsible for interstitial fibrosis in PKD (52, 53). Consistent with the reduced collagen deposition, FTY720 treatment markedly decreased the presence of myofibroblasts of PKD-PKCζ^+/+^ mice, whereas the drug had no significant effect on PKD-PKCζ^−/−^ mice (Fig. 4*E*). Macrophage accumulation has been shown to contribute to PKD progression (54–57). Probing with the macrophage marker F4/80 revealed that renal macrophages were significantly reduced in PKD-PKCζ^+/+^ mice treated with FTY720, compared with vehicle, but not significantly in PKD-PKCζ^−/−^ mice (Fig. 4*F*). Terminal deoxynucleotidyl transferase dUTP nick-end labeling (TUNEL) assay was also significantly reduced in treated PKD-PKCζ^+/+^ mice, while a significant effect was not observed in PKD-PKCζ^−/−^ mice, suggesting that FTY720 suppresses apoptosis in a PKCζ-specific manner in this model (Fig. 4*G*). The cell cycle marker *K*_i_-67 was also significantly reduced in cyst-lining cells of PKD-PKCζ^+/+^ mice in response to treatment, indicating a suppression of cell proliferation, and was not significantly affected in PKD-PKCζ^−/−^ mice (Fig. 4*H*). Last, immune staining demonstrated a pronounced reduction in STAT3 activity (tyrosine 705 phosphorylation) in FTY720-treated PKD-PKCζ^+/+^ mice, compared with vehicle control (Fig. 4*I*). The reduction in STAT3 activity was eliminated when PKCζ is knocked out. Overall, statistical comparisons between treated cystic PKCζ^+/+^ and PKCζ^−/−^ groups revealed PKCζ-dependent differences in several metrics of PKD disease progression upon treatment with FTY720, suggesting that PKCζ is involved in these drug effects.

**Fig. 5.**
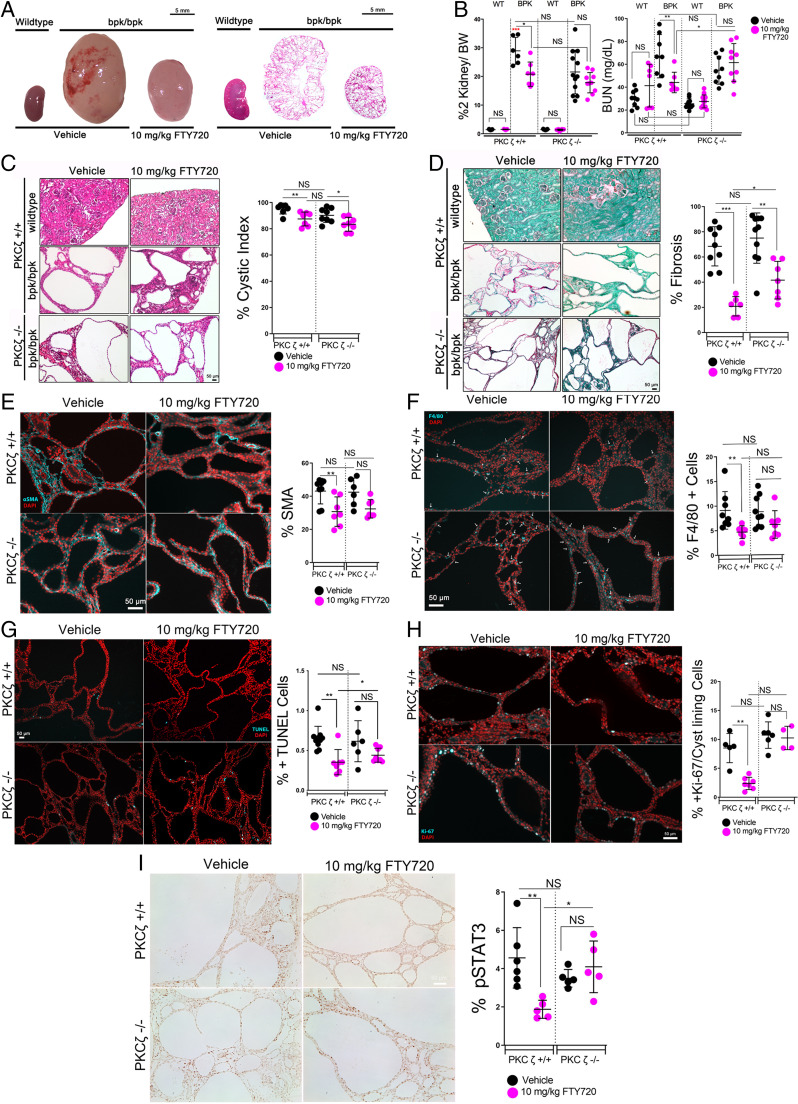
FTY720 improves disease progression in the bpk mouse model, while the improvement is diminished in bpk PKCζ-knockout mice. Wild-type (WT) and bpk/bpk (bpk) mice and wild-type and bpk mice crossed into a PKCζ-null model were treated with daily IP injections of 10 mg/kg FTY720 or vehicle control (2% DMSO) from days 7 to 16 and assessed for disease progression. (*A*) Representative gross kidney images of wild-type and bpk vehicle or FTY720 treated (*Left*), and representative 4× images of full-kidney H&E-stained kidney sections (*Right*). (*B*) Two-kidney to body weight (% 2K/BW) (*Left*) and BUN (*Right*). Red asterisk represents end-stage renal failure. (*C*) Representative images of H&E-stained wild-type, bpk, and bpk PKCζ-null mouse kidneys and percent cystic index, (*D*) Sirius red and fast-green collagen staining of bpk mouse kidneys and percent fibrosis. € α-SMA immunofluorescence of bpk or bpk PKCζ-knockout mice kidneys treated with 10 mg/kg FTY720 or vehicle injection and quantification. (*F*) Macrophage marker F4/80 immunofluorescence of bpk or bpk PKCζ-knockout mice kidneys treated with 10 mg/kg FTY720 or vehicle injection and quantification. (*G*) TUNEL assay for apoptosis of bpk and bpk PKCζ-null kidney sections and percentage of TUNEL-positive cells. (*H*) *K*_i_-67 cellular proliferation marker immunofluorescence stain and quantification performed by counting the cyst-lining–positive *K*_i_-67 cells per total cyst-lining cells. (*I*) Immunohistochemical stain for phospho-STAT3 (Y705) of bpk and bpk PKCζ-null kidney sections and quantification. Scale bars, 50 µm. *P < 0.05; **P < 0.01; ***P < 0.001.

We also treated the nonorthologous bpk mouse model with daily IP injections of 10 mg/kg FTY720 from postnatal days 7 to 16 and found a similar attenuation of cyst progression in treated mice compared with vehicle controls, most of which appeared to be PKCζ dependent. We identified significant reductions in gross kidney size (Fig. 5*A*), two-kidney to body weight ratio, and BUN concentration (Fig. 5*B*) in cystic (bpk/bpk) PKCζ^+/+^ mice treated with FTY720. Furthermore, cystic PKCζ^+/+^ mice also showed significant improvements in cystic index (Fig. 5*C*), fibrosis (Fig. 5*D*), numbers of myofibroblasts and macrophages (Fig. 5*E* and *F*), apoptosis (Fig. 5*G*), proliferation (Fig. 5*H*), and pSTAT3 (Fig. 5*I*) in kidneys treated with FTY720, compared with vehicle controls. Statistical comparisons of the treated cystic PKCζ^+/+^ and PKCζ^−/−^ groups revealed significant differences for BUN, fibrosis, apoptosis, proliferation, and pSTAT3 in response to FTY720 treatment that were dependent on PKCζ expression in the bpk mouse model. Just as in the PKD model, the most prominent differences between PKCζ^+/+^ and PKCζ^−/−^ cystic mice treated with FTY720 were in BUN concentration, fibrosis, and STAT3 activation. This suggests that the improvements observed in these markers of PKD progression may be the most dependent on PKCζ function. Notably, however, treatment with FTY720 also resulted in some PKCζ-independent improvements in the two models of PKD. PKCζ-independent benefits of FTY720 treatment may be due to the drug’s known effects on other antiinflammatory, antifibrotic, or S1P inhibitory pathways (58–64).

Taken together, these data demonstrate that PKCζ dysregulation plays a prominent role in PKD disease progression in orthologous and nonorthologous mouse models and that many of the beneficial effects of FTY720 in the disease are specific to its effect on PKCζ.

## Discussion

Here we report that PC1 interacts with and enhances PKCζ expression, that PC1 is a phosphorylation target of PKCζ in vitro, and that PKCζ expression is decreased in the kidneys of patients with ADPKD as well as in animal models of PKD. We found that PC1 increases PKCζ expression, particularly at cell junctions of cultured epithelial cells, and our results expand on the previous findings of Castelli et al. (30) by demonstrating that PC1 is a phospho-target of PKCζ and identifying several phosphorylation sites in the cytosolic tail of PC1. Finally, we identify an FDA-approved therapeutic, FTY720, that coordinately rescues PKCζ function in PKD mouse models while attenuating the disease.

Castelli et al. (30) reported preferential formation of a par3-aPKC complex in PC1-expressing cells and preferential formation of a par6-aPKC complex in PC1-null cells. They reported that aPKC was active in these complexes and that aPKC activity was necessary for directional migration of kidney epithelial cells. We find, using more isoform-selective antibodies (65), that PKCζ expression levels reduce while PKCι/λ levels increase in PC-1 knockdown cells (*SI Appendix*, Fig. S4*A*) and in kidneys of orthologous Pkd1^cond/−^ mice (Fig. 2). These data suggest that PC1 may promote the formation of par3-PKCζ complexes instead of par6-PKCι/λ complexes. However, additional research will be necessary to further elucidate how PC1 regulates these PAR polarity complexes, as well as their potential impact on the Crumbs and Scribble polarity complexes. Our results expand on previous findings demonstrating that PC1 is a phospho-target of PKCζ with several phosphorylation sites in the cytosolic tail. These sites may play a role in the control of cell migration and convergent extension of renal epithelial cells; however, this remains to be investigated.

We also demonstrated that restoration of PKCζ function with the FDA-approved, immunomodulatory drug FTY720 improves numerous markers of PKD progression in multiple mouse models (Figs. 4 and 5). Importantly, the beneficial effects of FTY720 were largely eliminated in animals lacking PKCζ expression. In particular, we observed substantial inhibition of STAT3 activity with FTY720 treatment that was dependent on PKCζ expression (Figs. 4*I* and 5*I*). A recent study independently reported similar STAT3 inhibition and overall reduced cyst growth with FTY720 treatment in the Cy/+ Han:SPRD rat model (62). These authors attributed this finding to the drug’s inhibition of the S1P receptor, subsequent inhibition of the proinflammatory pathways of STAT3 and NF-κB, and their downstream inflammatory cytokines IL6 and TNFα (62). This reported effect of FTY720 on STAT3 activity and inflammation is consistent with our results. However, our finding that the introduction of a PKCζ knockout eliminates both the reduction in STAT3 activity and the improvement in disease progression suggests that the benefit of FTY720 treatment in the models we tested is likely more dependent on its modulation of PKCζ activity rather than its inhibition of the S1P receptor per se.

We have previously reported that PC1 and its C-terminal fragments activate STAT3 through a dual mechanism, potentially explaining STAT3 activation in patients who overexpress STAT3-activating PC1 fragments (15, 16). However, STAT3 activity is also up-regulated in mouse models of PKD that lack PC1 expression, suggesting that STAT3 activation in PKD is controlled by a complex set of regulatory pathways (11). Our results here suggest that STAT3 activity is negatively regulated by PKCζ in two mouse models of PKD. Our finding in the orthologous Pkd1^cond/−^ model suggests that PKCζ inhibition of STAT3 activation is caused by a lack of PC1 expression. However, we also observe similar PKCζ-dependent STAT3 activity in the bpk mouse model, a model which expresses PC1 (66). Taken together, these data suggest that PKCζ is an important regulator of STAT3 activity in PKD, regardless of PC1-expression status, and thus broadens its value as a therapeutic target in multiple forms of PKD.

FTY720 may also improve disease progression by recovering other pathways dysregulated in PKD. PC1-null cells exhibit metabolic features similar to the Warburg effect, and recent evidence suggests that metabolic reprogramming plays an important role in PKD progression (67, 68). It has been shown that PKCζ deficiency promotes the metabolic shift required for malignant cells to utilize glutamine in the absence of glucose, thus allowing proliferation in the absence of traditional nutrients. Our group recently reported that this metabolic inflexibility can be exploited for an effective therapy by inducing the state of ketosis (24). We show here a PKCζ-dependent decrease in proliferation observed in treated mouse models of PKD, suggesting that restoration of PKCζ’s function with FTY720 may reverse elements of the Warburg effect and reduce the proliferation advantage conferred by loss of PC1 in kidney cells. Separately, there is growing evidence that metformin may be beneficial in PKD through its activation of AMPK (69, 70). Given the fact that metformin-induced AMPK activation requires PKCζ to activate LKB1 (37), it is tempting to speculate that FTY720 may indirectly activate AMPK through its modulation of PKCζ function and attenuate ADPKD progression in a similar manner as metformin.

FTY720 may also improve PKD progression by regulating PKCζ-independent pathways, as evidenced by the select improvements in markers of disease progression that we observed in the absence of PKCζ. The drug has been shown to activate autophagy (71), which is thought to be suppressed in PKD (72), and there is evidence that FTY720 attenuates kidney fibrosis and down-regulates a wide variety of inflammatory pathways (58–61), both of which are known to occur in PKD (73, 74). These studies, along with our findings, corroborate FTY720 as a promising therapeutic for the treatment of PKD.

Overall, this study demonstrates that PKCζ dysregulation is of central importance in the pathogenesis of PKD and that this pathway represents a target for pharmacological intervention.

## Materials and Methods

### Animal Studies.

All animal studies were performed in accordance with the rules and regulations of the NIH with approval of the University of California, Santa Barbara, Institutional Animal Care and Use Committee. Mice were maintained in standard vivarium conditions. The bpk/bpk mouse strain was a contribution from Oliver Wessely at the Cleveland Clinic. The Pkd1 Pkd1^cond/cond^:NesCre (Pkd1^cond/cond)^ and Pkd1^cond/−^:NesCre (Pkd1^cond/−^) mouse models were described previously (19, 20). PKCζ transgenic knockout mice (PKCζ^−/−^), contributed by Jorge Moscat at Weill Cornell Medicine, were crossed into the bpk/bpk and Pkd1^cond/−^ mouse models. All mouse models were on a C57/Bl6 genetic background. PKCζ^−/−^ mice were genotyped by PCR for the presence of PKCζ (forward primer: GCTCCTCCATCACCATGCTT; reverse primer: TGAGCACACGGAAGGAAGTC; 263-bp product) or the NeoR cassette (forward primer: CAGACAATCGGCTGCTCTGA; reverse primer: CCCTGATGCTCTTCGTCCAG; 413-bp product). Mice were treated with FTY720 via IP injection every 24 h. FTY720 (fingolimod) was obtained from Cayman Chemical Company (Ann Arbor, MI), reconstituted in dimethyl sulfoxide (DMSO), and diluted in sterile water to a final concentration of 2% DMSO. The standard IP injection volume was 10 μL/g mouse body weight. For studies involving the Pkd1^cond/−^ mouse model, injections were performed from postnatal days 7 to 20. Mice were euthanized on day 21. For the nonorthologous bpk model, injections were performed from postnatal day 7 to day 16. Mice were euthanized on day 17. For single-injection studies, mice were injected with FTY720 IP at the indicated dosages and euthanized at the indicated time points following injection. Mice were euthanized using a ketamine/xylazine solution, diluted in sterile saline solution.

### Mouse Kidney Section Histology and Immunostaining.

Formalin-fixed, paraffin-embedded (FFPE) tissues were sectioned (5 μm) and processed for H&E, immunofluorescence, or immunohistochemical staining, as described previously (75). For immunofluorescence costaining of PKCζ and DBA, standard 5-μm FFPE sections underwent epitope retrieval by pressure cooking in Tris-ethylenediaminetetraacetic acid (EDTA) at pH 9 (10 mM Tris base, 1 mM EDTA). Sections were blocked in 5% goat serum, 0.1% TX-100 in Tris-buffered saline plus 0.1% Tween-20. Primary antibody staining for PKCζ (C24E6) rabbit monoclonal antibody (Cell Signaling) and rhodamine-labeled DBA (Vector Laboratories) were carried out as described previously (76). For α-smooth muscle actin (α-SMA; Abcam) and *K*_i_-67 (Millipore) staining, 5-μm FFPE sections were subjected to antigen retrieval by pressure cooking in 10 mM sodium citrate at pH 6, as previously described (24). For F4/80 (ThermoFisher) and phospho-STAT3 (Y705) (Cell Signaling) staining, 5-μm FFPE sections underwent enzymatic epitope retrieval using 20 μg/mL of Proteinase K (Sigma Aldrich) diluted in TE buffer (50 mM Tris base, 1 mM EDTA, pH 8). Sections were incubated with blocking buffer (10% goat serum, 1% bovine serum albumin, 0.5% gelatin in Tris-buffered saline with 0.05% Tween-20), followed by primary antibody. For phospho-STAT3 (Y705) staining, endogenous peroxidase activity was blocked using 3% hydrogen peroxide in Tris-buffered saline. Primary antibody was detected using goat anti-rabbit horseradish peroxidase (Jackson ImmunoResearch) and the DAB-Substrate Kit (Vector Laboratories). Sections were treated with 0.1% Sudan Black B in 70% ethanol for 20 min to quench autofluorescence prior to secondary incubation. Sections were then incubated with species-specific fluorescently labeled secondary antibodies, diluted 1:200 in blocking buffer for 1 h. The following secondary antibodies were used for immunostaining: Alexa Fluor 594 goat anti–rabbit immunoglobulin G (IgG; Invitrogen), Alexa Fluor 488 donkey anti–rabbit IgG (H+L; ThermoFisher), and Alexa Fluor 488 goat anti–rat IgG (Invitrogen). All sections were washed in tris-buffered saline plus Tween, stained with DAPI for 10 min at 25 °C, and mounted on slides using ProLong Gold Antifade Reagent (Life Technologies) following secondary incubation. The DeadEnd Fluorometric TUNEL system (Promega) was used for TUNEL staining and performed as specified by the manufacturer.

### Cystic Index and Fibrosis Quantification.

Tissue sections were stained with H&E and micrographs acquired. To quantify cystic index, images were overlaid with a grid in Adobe Photoshop and intersecting points on cysts or normal tissue were counted manually. To determine fibrosis, the Sirius Red/Fast Green Collagen Staining Kit was used per assay procedure (#9046, Chrondrex, Inc.) and images were obtained for quantification by grid overlay. Intersecting points on Sirius red–positive areas were counted excluding blood vessels and cysts over the total number of intersections.

### Myofibroblast and Macrophage Quantification.

To estimate the prevalence of renal myofibroblasts and macrophages, kidney sections were stained for α-SMA or F4/80, respectively. Grid intersection points on α-SMA or F4/80-positive structures were counted and divided by the total intersections, excluding nontissue intersections, and expressed as a percentage.

### *K*_i_-67 Quantification.

*K*_i_-67–immunostained kidney sections were imaged and the number of cyst-lining cells expressing the cell cycle marker *K*_i_-67 were counted and expressed as a fraction of the total cyst-lining cells. Approximately 1,000 cyst-lining cells were counted per kidney section.

### TUNEL and Phospho-STAT3 Quantification.

TUNEL and pSTAT3-stained sections were imaged and the total number of DAPI-positive nuclei were analyzed using FIJI image-processing software (ImageJ; NIH). At least 10 areas per kidney were imaged and the number of either TUNEL- or pSTAT3-positive cells was counted. Percentages of TUNEL- or pSTAT3-positive cells are expressed as a fraction of the total counted nuclei.

### Blood Urea Nitrogen.

Blood was extracted via cardiac puncture at the time of euthanasia. Serum was separated using BD Microtainer serum separators and frozen. BUN concentration was quantified using the Urea Nitrogen Colorimetric Detection Kit (Invitrogen, EIABUN) according to the kit procedure.

### Cell Culture and Transfection.

HEK293T cells were cultured at 37 °C in Dulbecco’s modified Eagle’s medium (Cellgro) supplemented with 10% heat-inactivated fetal bovine serum (Omega Scientific), and 1× penicillin/streptomycin (Cellgro). MDCK cells were cultured at 37 °C in MEM (Cellgro) supplemented with 5% heat-inactivated fetal bovine serum, 1× penicillin/streptomycin, and 1× l-glutamine (Cellgro). RPE1 cells were cultured at 37 °C in Dulbecco’s modified Eagle’s medium–reduced serum (Cellgro) supplemented with 4% heat-inactivated fetal bovine serum, 1× penicillin/streptomycin, and 0.01 mg/mL hygromycin (Thermo/Fisher Scientific). Transient transfections of HEK293T cells were performed using Lipofectamine 2000 (Life Technologies) or Turbofect (Thermo Scientific) per the manufacture’s protocol.

### Plasmids.

Several PC1 cytoplasmic tail constructs in the plasmid pcDNA4/TO/Myc-His have been previously described (77). Additional PC1 deletion and phosphorylation mutants were made by site-directed mutagenesis and cloned into the pcDNA4/TO/Myc-His backbone. Constructs for PC1-membrane anchored fragments P100 and CTF were a gift from Feng Qian (Johns Hopkins School of Medicine). Lentiviral PC1–shRNA constructs were gifts from Gabriele Gusella (Mount Sinai, NY) (78). The STAT3 luciferase reporter, containing four γ-interferon activation–site elements upstream of the luciferase gene and the AP1 luciferase reporter, containing enhancer element sequence for cJun, cFos, and ATF, have been described previously (15, 16).The pGEX-MARCKS(96-184) plasmid was a gift from Jae-Won Soh (Inha University, Korea). FLAG.PKC**ζ** plasmid was a gift from Alex Toker (Addgene plasmid #10799; http://n2t.net/addgene:10799; RRID:Addgene_10799) (79). pLTR PKCδ was a gift from Frederic Mushinski (Addgene plasmid #8419; http://n2t.net/addgene:8419; RRID:Addgene_8419) (80) and was recloned into pcDNA4/HA by restriction digest.

### Antibodies.

Anti-PC1 antibodies have been previously described (77). Anti-PKCδ (C-20), anti-PC2, anti-STAT3, and anti-STAT3 (Y705) were from Santa Cruz Biotechnology. Anti-GST and anti-actin were from Sigma Aldrich. Anti-myc (9E10) hybridoma cells were from Bioworld Technology and were used to produce antibody. Anti-PKCλ was acquired from BD Biosciences. Anti-PKCζ/λ (pT555/563) was obtained from Invitrogen. Anti-PKCζ/λ (pT410/403), anti-PKCζ (C24E6), anti-LC3, anti-HA, and anti-FLAG antibodies were acquired from Cell Signaling Technologies.

### Immunoprecipitation.

Cells were washed in cold phosphate-buffered saline and scraped in lysis buffer (10 mM Tris⋅HCl at pH 7.4, 150 mM NaCl, and 1% Triton X-100) containing protease inhibitors and a phosphatase inhibitor mixture (Sigma). Lysates were rotated at 4 °C for 30 min. Protein-A-Sepharose beads (Amersham, preblocked with 1% bovine serum albumin) were coated with either anti-PKC**ζ** antibody or control IgG overnight. Precleared lysates were then incubated with coated Protein A beads at 4 °C for 1 h. The beads were washed with lysis buffer two times and samples were analyzed by Western blotting with the indicated antibodies.

For GST pull-downs, precleared lysates were incubated with glutathione beads (preblocked with 1% fetal bovine serum) (Omega Scientific) at 4 °C for 1 h. The beads were washed with lysis buffer two times and samples were analyzed by Western blotting with the indicated antibodies.

### In Vitro Phosphorylation Assay.

GST-tagged PC1 expression constructs were expressed and purified with glutathione beads as described above. Beads were washed once with lysis buffer, twice with wash buffer (25 mM Tris⋅HCl at pH 7.4, 0.05% Triton X-100, 1 mM CaCl_2_, 20 mM MgCl_2_, 1 mM dithiothreitol [DTT]), and then resuspended in 50 μL of kinase reaction buffer per reaction (25 mM Tris⋅HCl at pH 7.4, 0.05% Triton X-100, 1 mM CaCl_2_, 20 mM MgCl_2_, 1 mM DTT, 100 μM adenosine triphosphate [ATP], 0.2 mg/mL phosphatidyl serine, 200 nM phorbol-12-myristate-13-acetate, 300 μCi/mL ATP). Beads were incubated for 45 min at 30 °C with either 20 ng of recombinant PKCδ (Calbiochem) or 40 ng PKCζ (Sigma). The reactions were stopped by washing the beads once with ice-cold wash buffer. Phosphorylated proteins were resolved by sodium dodecyl sulfate–polyacrylamide gel electrophoresis. The gel was dried for 6 h using a gel dryer (Bio-Rad), and radioactive phosphate was detected using a phosphorimager (Bio-Rad). Protein loading of each reaction was determined in parallel by Western blot analysis. All experiments are representative of three independent experiments.

### Human Kidney Samples.

Tissue samples from anonymous patients with ADPKD or normal controls were obtained from the National Disease Research Interchange, per institutional guidelines. Samples were frozen in liquid nitrogen and then cryopulverized using a mortar and pestle. Fine shavings of tissue were lysed in sodium dodecyl sulfate sample buffer (lacking bromophenol blue) and were quantified by A280. Normalized samples were used for Western blot analysis.

### Mass Spectrometry.

#### Phosphopeptide enrichment.

HEK293T cells were transfected with 5 µg of DNA for each construct using the calcium phosphate transfection method. After 2 d, cells were harvested in ice-cold lysis buffer containing 8M urea, 50 mM ammonium bicarbonate, and Halt protease phosphatase inhibitor mixture (Pierce). Phosphopeptides were enriched as previously described (81, 82), with slight modifications. Briefly, proteins were reduced using 10 mM DTT and subsequently alkylated using 20 mM iodoacetamide. Remaining iodoacetamide was quenched by adding additional DTT at a final concentration of 20 mM. Peptides were digested overnight (16 h) at room temperature with shaking (200 rpm) using trypsin (1:50 weight per weight as a protease). Peptides were desalted using Oasis HLB columns (Waters). After drying down in a SpeedVac (Christ), peptides were resuspended in 5% acetic acid. Phosphopeptides were enriched using Fe-NTA IMAC resin columns (Pierce). Eluted phosphopeptides were dried and were subsequently cleaned using C18 ZipTips. Eluted phosphopeptides were dried and resuspended in 0.1% formic acid for final analysis.

#### Nano-liquid chromatography–tandem mass spectrometry.

Peptides were separated by nano-liquid chromatography and were analyzed on a LTQ Orbitrap Discovery mass spectrometer using the exact same chemicals, instrumental setup, and settings as previously described (83).

#### Bioinformatic analysis.

RAW files were analyzed using the Sequest search algorithm implemented into the Proteome Discoverer environment (version 1.4). The database used was human (reference proteome, obtained from Uniprot in January 2013, no isoforms). Settings for identification were as previously described (81). The false discovery rate for peptide identification was set to 0.01. Only phosphorylation sites with a localization probability of 0.75 (phosphoRS score) were accepted. Fragment ion-match tolerance was 0.5 Da, and parent ion-match tolerance was 20 ppm. Phosphorylation of serines, threonines, and tyrosines was used as a variable modification, whereas alkylation of cysteine was used as a fixed modification. Quantitation and visualization of precursor ion chromatograms were performed using the NHBLI Quoil software in the label-free quantification modus (84). Within the quantification, a maximum retention time difference of 1 min and a mass accuracy tolerance of 20 ppm was allowed.

### Statistical Analysis.

Following tests for normal distribution, the statistical analyses were performed using Mann–Whitney unpaired one-tailed Student’s *t* test. Analysis was performed using Prism software (GraphPad). Animals with PKD were housed with litter mates in groups of two to four animals without regard to genotype, resulting in a random distribution of PKD and wild-type animals in cohorts. Experimenters were not blinded to the treatment of genotypes of animals. The analysis of collagen, cystic index, α-SMA, macrophage TUNEL, Ki67, and STAT3 images was conducted blinded. Exclusion criteria were based upon animal well being. No animals were excluded from this study. No power analysis was done to determine sample sizes. Sample sizes were chosen based on experience with previous studies with animals with PKD in our laboratory.

## Supplementary Material

Supplementary File

## Data Availability

All study data are included in the article and/or supporting information.
