# Supplementary material for: Restoration of atypical protein kinase C ζ function in autosomal dominant polycystic kidney disease ameliorates disease progression
Source: Proc Natl Acad Sci U S A. 2022 Jul 22;119(30):e2121267119. doi: 10.1073/pnas.2121267119 (PMC9335328; doi:10.1073/pnas.2121267119)
Supplement: Supplementary File [file pnas.2121267119.sapp.pdf]

PNAS
www.pnas.org

Supplementary Information for

Restoration of Atypical Protein Kinase C ζ Function in Autosomal Dominant Polycystic Kidney Disease Ameliorates Disease Progression

Masaw Akbari[a, 1], Jonathan D. West[a, 1], Nicholas Doerr[a, 1], Kevin R. Kipp[a], Neda Marhamati[a], Sabrina Vuong[a], Yidi Wang[a,d], Markus M. Rinschen[b,e], Jeff Talbot[a], Oliver Wessely[c], and Thomas Weimbs[a,2]

Corresponding author: Thomas Weimbs
Email: weimbs@ucsb.edu

This PDF file includes:

Supplementary text
Figures S1 to S7

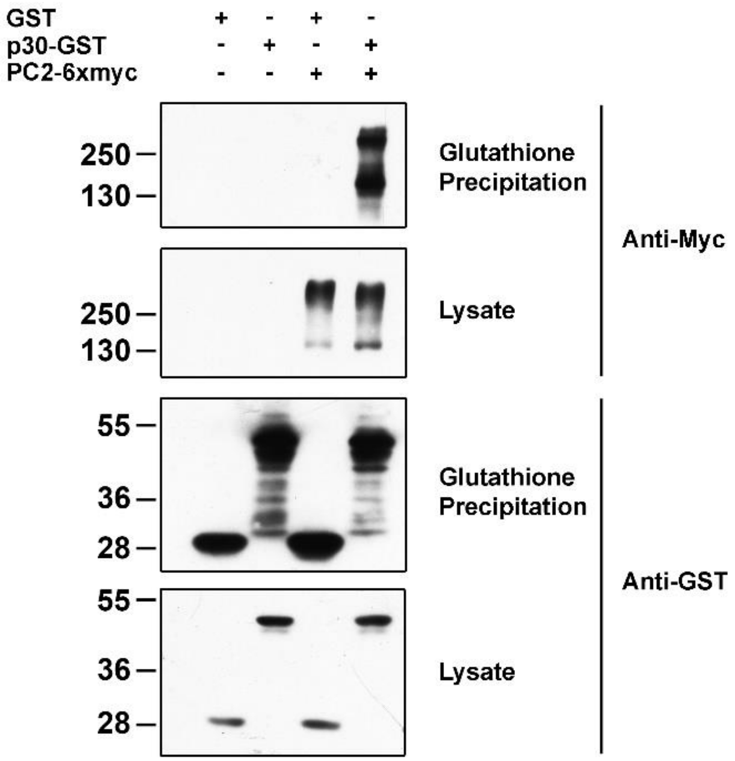

Fig. S1. GST-tagged PC1 interacts with PC2. HEK293T cells were transfected with GST, p30-GST, or PC2-6xmyc. GST-tagged proteins were precipitated from lysates with glutathione-agarose. Lysates and precipitates were analyzed by western blot using the specified antibodies.

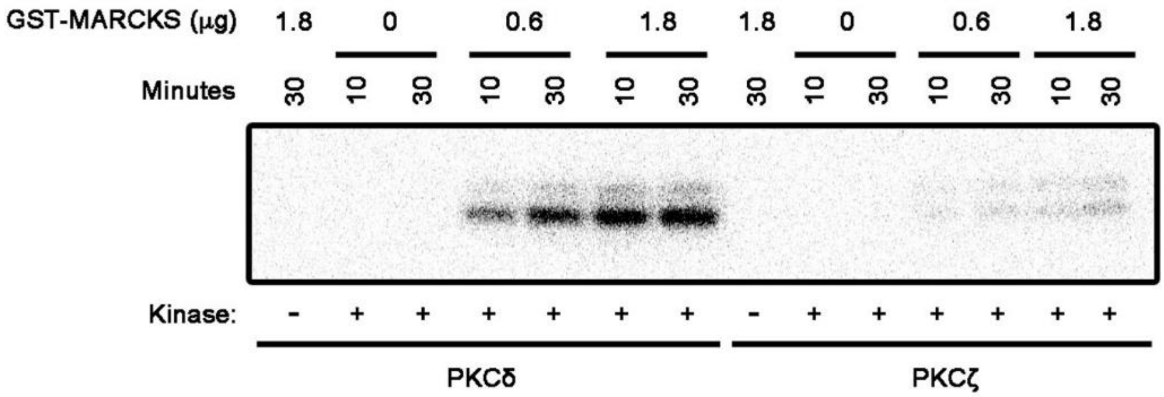

Fig. S2. GST-MARCKS is a substrate of PKCδ and PKCζ. Indicated amounts of recombinant GST-MARCKS protein was bound to glutathione-agarose and incubated with 20ng recombinant PKCδ or PKCζ for 10 or 30 minutes at 37°C. Phosphorylated proteins were resolved by SDS-PAGE and detected by autoradiography.

A.

Serine	NetphosK1.0	PKCd	PKCz
**4144**	---	4	5
**4165**	---	27	12
**4166**	0.69	20	16
**4251**	0.56	1	0.2
**4258**	0.63	24	7
**4259**	0.62	51	12
**4263**	---	63	26
**4272**	---	13	21
**4278**	---	4	1

B.

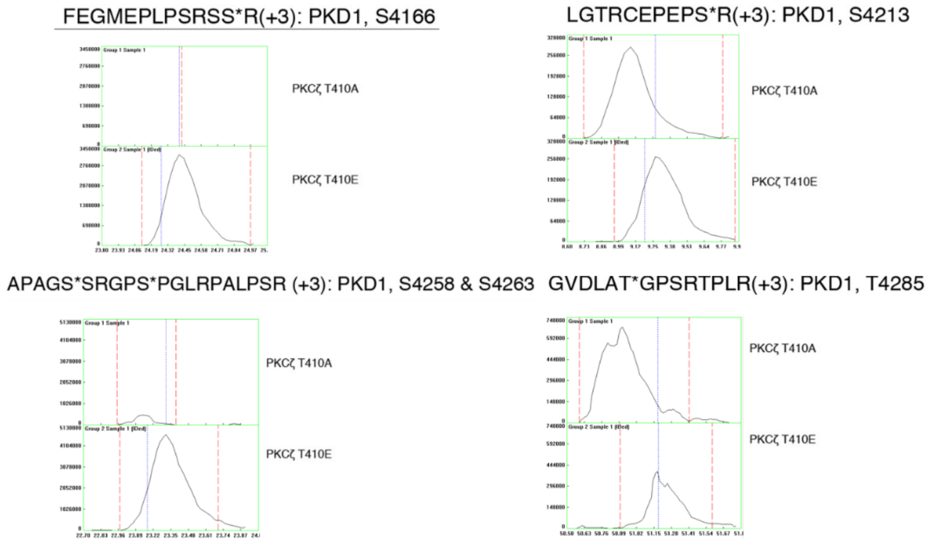

C.

Peptide	Residues	m/z	PKCζ T410A (Area*1E5)	PKCζ T410E (Area*1E5)
FEGMEPLPSRSSR (+3)	S4165 or S4166	524.9	0	92
LGTRCEPEPSR (+3)	S4213	461.21	9	9
APAGSSRGPSPGLRPALPSR (+3)	S4258 or S4259, S4263	697.67	7	144
GVDLATGPSRTPLR (+3)	T4285	507.26	23	25

Fig. S3. The PC1 tail is a predicted target of PKC phosphorylation. (A) Scores assigned to individual serines within the PC1 tail by NetphosK1.0 (0=low, 1=high) or a tool by Fujii et al (0=high, 100=low), based on their likelihood to be phosphorylated by PKCs (NetphosK1.0) or specific isoforms PKCδ and PKCζ (Fujii). (B) HEK293T cells were transfected with PC1-p30 and either inactive PKCζ (T410A) or active PKCζ (T410E). Phosphopeptides enriched from lysates were quantified by mass spectrometry. (C) Table summarizing PC1 phospho-peptides identified by quantitative mass spec from lysates of HEK293T cells transiently co-expressing PC1-p30 and either kinase dead PKCζ (T410A) or constitutive active PKCζ (T410E).

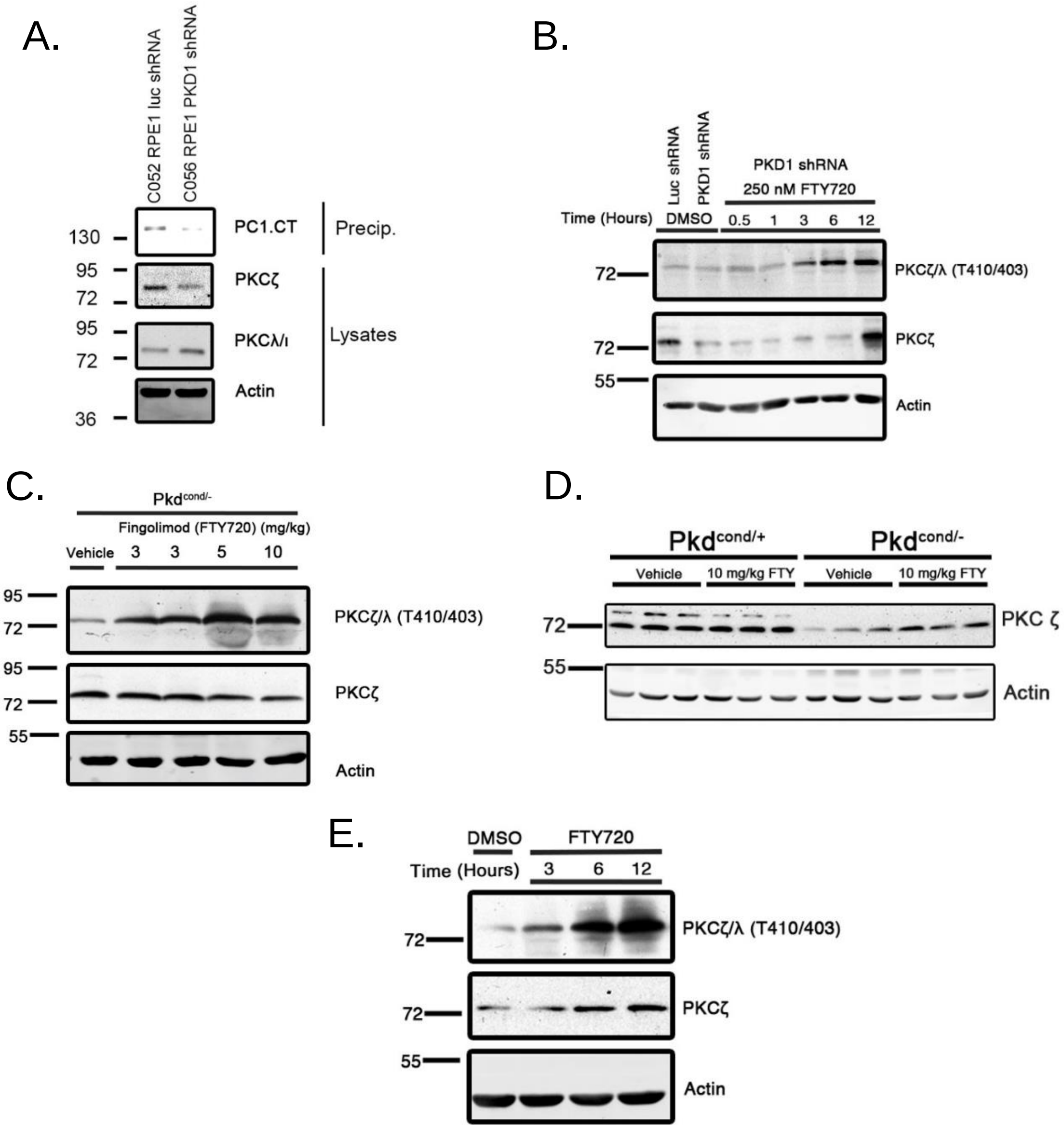

Fig. S4. FTY720 activates PKCζ *in vitro* and *in vivo*. (A) Confirmation of stable transfection of Pkd1 shRNA knockdown in RPE1 cells (Pkd1 shRNA) and scramble control (Luc shRNA). Cell lysates were precipitated with an anti-PC1 antibody and analyzed by western blot. (B) Western blot of RPE1 cells with a stable shRNA knockdown of either Pkd1 (Pkd1 shRNA) or scramble control (Luc shRNA), treated with 250 nM FTY720 over a time course. (C) Western blot of kidney tissue lysates from cystic (Pkd1$^{cond/-}$) mice injected with a single dose of 3, 5 or 10 mg/kg FTY720, compared to vehicle controls. Mice were euthanized 12 hours after IP injection. (D) Cystic and wildtype PKD mice treated with daily IP injections of 10 mg/kg FTY720 or vehicle from postnatal day 7 to 20 tissue was harvested 12 hours after the last injection and kidney lysates were analyzed by western blot. (E) Western blot of MDCK cells treated with 1 μM FTY720 or DMSO control over a time course of 3, 6 or 12 hours.

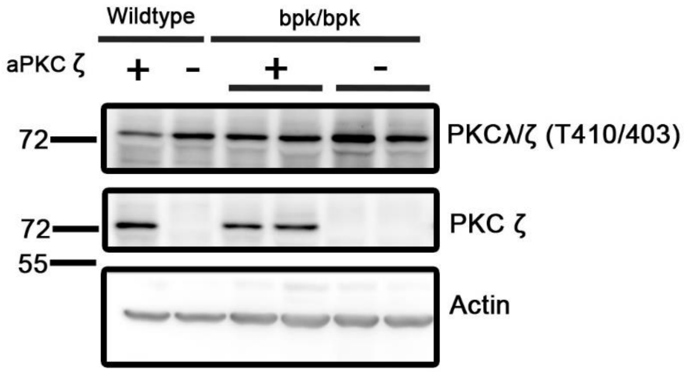

Fig. S5. aPKC activation in PKCζ knockout bpk mice. Western blot of kidney tissue lysates from bpk or wildtype mice that have a transgenic knockout of PKCζ compared to PKCζ +/+ bpk mice express comparable activation at the Threonine 410/403 site, indicating a potential compensatory mechanism between ζ and λ phospho-sites.

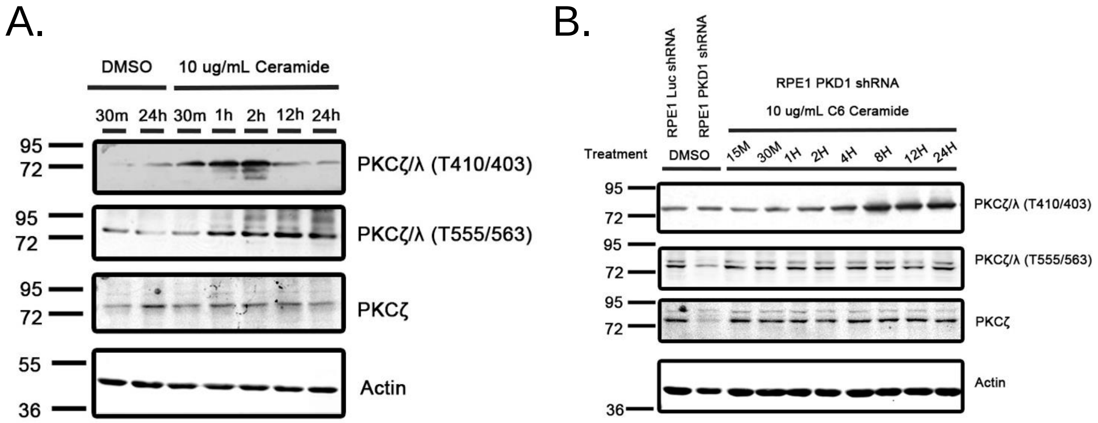

Fig. S6. Ceramide activates PKCζ in Kidney cells and *in vitro* model of PKD (A) IMCD cells treated with DMSO or Ceramide for 30 minutes, 1 hour, 2 hours, 12 hours or 24 hours. (B) Western blot of RPE1 cells with a stable shRNA knockdown of either Pkd1 (Pkd1 shRNA) or scramble control (Luc shRNA), treated with 10 ug/mL of C6 Ceramide over a time course treatment.

A.

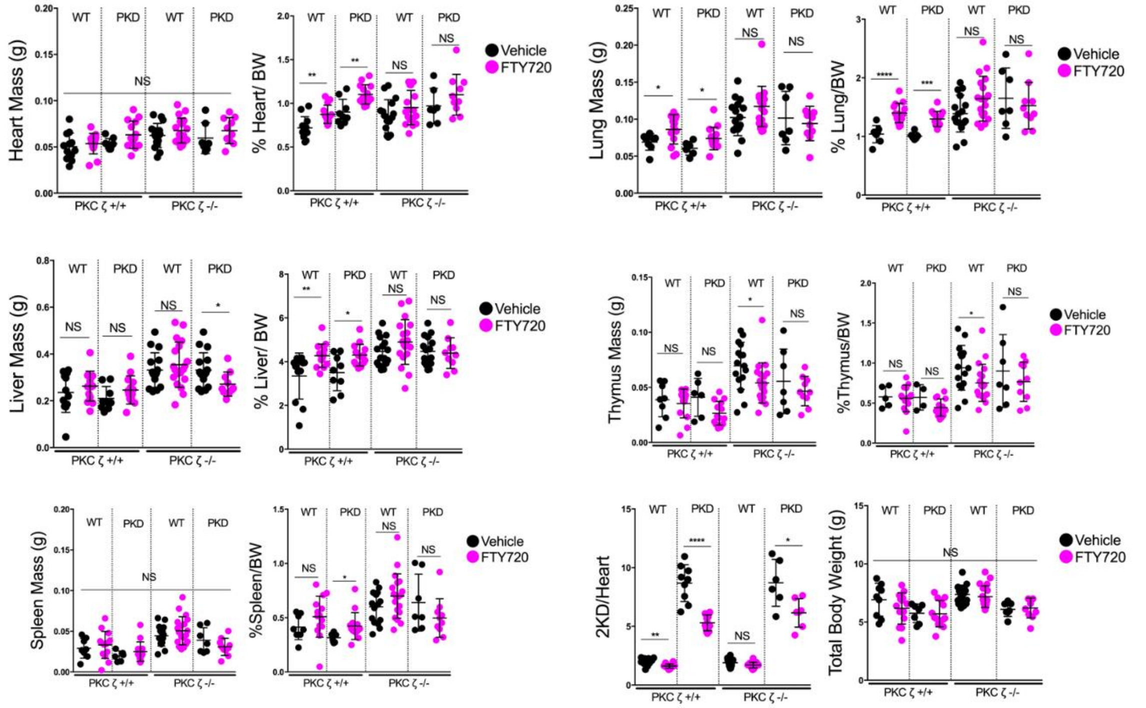

B.

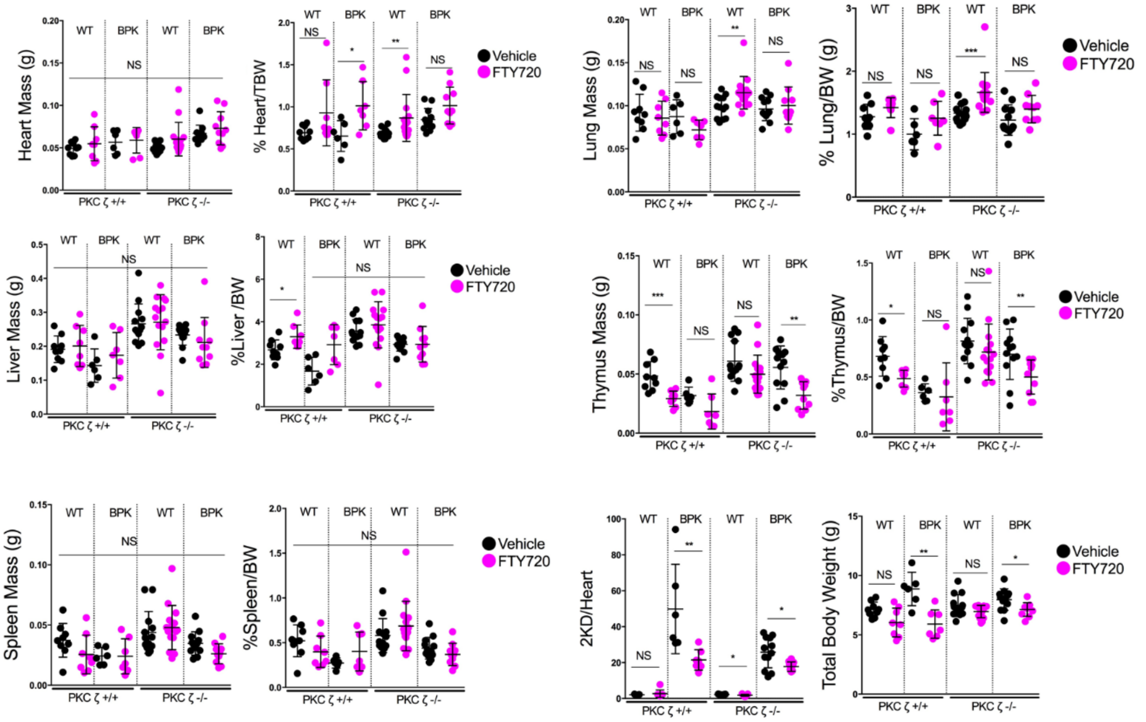

Fig. S7. Pkd1$^{cond/-}$ and bpk mouse models tissue weights from 10 mg/kg FTY720 treatment. Tissue weights of Pkd1$^{cond/-}$ (A) and bpk (B) mice treated with daily IP injections of 10 mg/kg FTY720. Statistical significance was determined using Mann-Whitney analysis.
